# Letter-, Word- and Mind-Blindness

**Published:** 1900-12

**Authors:** 


**Letter-, Word- and Mind-Blindness.** By James Hinshelwood, M.D. Pp. 88. London: H. K. Lewis. 1900.

The first chapter, an interesting essay on the visual memory, forms an introduction to the remaining four chapters, which originally appeared in the *Lancet*. The reader will find in them an excellent account of certain varieties of aphasia; the illustrative cases are well reported, and the meaning and mode of production of the symptoms fully discussed. Many readers, who read with interest the papers when they first appeared, will be glad to have them in this convenient form.

